# Classification of Complex Emotions Using EEG and Virtual Environment: Proof of Concept and Therapeutic Implication

**DOI:** 10.3389/fnhum.2021.711279

**Published:** 2021-08-26

**Authors:** Eleonora De Filippi, Mara Wolter, Bruno R. P. Melo, Carlos J. Tierra-Criollo, Tiago Bortolini, Gustavo Deco, Jorge Moll

**Affiliations:** ^1^Computational Neuroscience Group, Center for Brain and Cognition, Department of Information and Communication Technologies, Universitat Pompeu Fabra, Barcelona, Spain; ^2^Cognitive Neuroscience and Neuroinformatics Unit, D'Or Institute for Research and Education (IDOR), Rio de Janeiro, Brazil; ^3^Biomedical Engineering Program, Instituto Alberto Luiz Coimbra de Pós-Graduação e Pesquisa de Engenharia, Federal University of Rio de Janeiro, Rio de Janeiro, Brazil; ^4^Institució Catalana de la Recerca i Estudis Avançats, Barcelona, Spain; ^5^Department of Neuropsychology, Max Planck Institute for Human Cognitive and Brain Sciences, Leipzig, Germany; ^6^Turner Institute for Brain and Mental Health, Monash University, Melbourne, VIC, Australia; ^7^Scients Institute, Palo Alto, CA, United States

**Keywords:** emotions, electroencephalography, classification, machine-learning, neuro-feedback, multimodal virtual scenario

## Abstract

During the last decades, neurofeedback training for emotional self-regulation has received significant attention from scientific and clinical communities. Most studies have investigated emotions using functional magnetic resonance imaging (fMRI), including the real-time application in neurofeedback training. However, the electroencephalogram (EEG) is a more suitable tool for therapeutic application. Our study aims at establishing a method to classify discrete complex emotions (e.g., tenderness and anguish) elicited through a near-immersive scenario that can be later used for EEG-neurofeedback. EEG-based affective computing studies have mainly focused on emotion classification based on dimensions, commonly using passive elicitation through single-modality stimuli. Here, we integrated both passive and active elicitation methods. We recorded electrophysiological data during emotion-evoking trials, combining emotional self-induction with a multimodal virtual environment. We extracted correlational and time-frequency features, including frontal-alpha asymmetry (FAA), using Complex Morlet Wavelet convolution. Thinking about future real-time applications, we performed within-subject classification using 1-s windows as samples and we applied trial-specific cross-validation. We opted for a traditional machine-learning classifier with low computational complexity and sufficient validation in online settings, the Support Vector Machine. Results of individual-based cross-validation using the whole feature sets showed considerable between-subject variability. The individual accuracies ranged from 59.2 to 92.9% using time-frequency/FAA and 62.4 to 92.4% using correlational features. We found that features of the temporal, occipital, and left-frontal channels were the most discriminative between the two emotions. Our results show that the suggested pipeline is suitable for individual-based classification of discrete emotions, paving the way for future personalized EEG-neurofeedback training.

## 1. Introduction

“This world's anguish is no different from the love we insist on holding back,” wrote Aberjhani in the book “Elemental: The Power of Illuminated Love” published in 2008 with the prize-winner artist Luther E. Vann. A decade later, there is still much to discover about the neurophysiology of complex emotions, which, according to some, represent a combination of basic emotions (Barrett et al., 2007) or primary emotive states and cognitive components such as event-feature-emotion complexes (Moll et al., 2005, 2008). The main novelty of this study is that we tried to classify complex affective states, which have been rarely explored with the EEG. We focus on tenderness as an affiliative feeling because it is the basis for empathy and prosocial behavior (Eslinger, 1998; Zahn et al., 2009), associated with social bonding, care, and well-being. On the contrary, anguish reflects a negative state of mental suffering linked to social dysfunction, withdrawal, and poor mental health (Corbett, 2015).

Given the importance of affiliative emotions for a range of psychosocial processes (Baumeister and Leary, 1995), psychotherapeutic approaches have embraced the training of such emotions as part of the therapeutic process (Gilbert and Procter, 2006; Lutz et al., 2008; Germer and Siegel, 2012; Neff and Germer, 2013). Real-time fMRI-Neurofeedback (NFB) training, a type of closed-loop brain-computer interface (BCI), has already been explored concerning the self-regulation of complex emotional states. Recently, Lorenzetti et al. (2018) demonstrated that the self-regulation of anguish and tenderness is possible by targeting the septohypothalamic network, engaged in affiliative feelings (Moll et al., 2011, 2012). In their experiment, Lorenzetti and colleagues validated a new protocol combining NFB with a multimodal, immersive virtual environment BCI and musical excerpts as means to deliver feedback to participants (Lorenzetti et al., 2018). However, the fMRI comes with several disadvantages, such as the signal delay (Friston et al., 1994), the high scanning cost, and an environment that can be hostile and uncomfortable. In contrast, EEG offers a higher temporal resolution and a direct measure of information processing. It is cheaper and relatively simple to use, making it a more accessible clinical environment tool. Therefore, the purpose of our work is to validate the use of the EEG with the protocol developed in Lorenzetti et al. (2018) to classify complex emotions.

Another novelty here is that we employed a multisensory virtual-reality (VR) scenario with audio input to elicit complex emotions. Music has been proven to be an effective stimulus in evoking genuine feelings in the laboratory setting (Scherer, 2004; Ribeiro et al., 2019). Moreover, the employment of VR has largely increased in the last years, and its validity for BCI application has been demonstrated (Coogan and He, 2018). For example, the work of Johnson et al. (2018) showed the feasibility of using VR BCI-training for motor recovery after stroke. Other studies have shown that integrating heterogeneous sensory stimuli leads to improved BCI performance by enhancing brain patterns (Wang et al., 2017; Huang et al., 2019) and that using multiple sensory modalities can improve the individuals' engagement and motivation in psychological and cognitive interventions (Cho et al., 2002; Lécuyer et al., 2008; Kovacevic et al., 2015). Furthermore, the study conducted by Baños and colleagues in 2004 has pointed out the advantage of immersion by means of VR scenarios when eliciting emotions through simulation of real experiences (Baños et al., 2004). To the contrary, widely used research paradigms in affective computing and BCI have typically employed single-modality or non-immersive stimuli to elicit emotions, such as the international affective pictures (IAPS; Lang et al., 1997), music, or movie clips. The ability to evoke affective states reliably and ethically in an experimental setting remains a critical challenge in emotion-recognition research (Kory and D'Mello, 2015). Therefore, we also encouraged participants to use personalized “mantras” (i.e., short emotionally loaded sentences) to facilitate self-induced emotional states. This approach to emotion induction can be of great advantage when using neurofeedback as self-regulation training (Yankauer, 1999).

To date, research in emotion classification using EEG signals mostly focused on distinguishing emotion based on psychological models (Russell, 2003; Rubin and Talarico, 2009), primarily employing the “circumplex model” (Lang et al., 1993), which portrays different types of emotions in a two-dimensional space comprising valence and arousal. Another model, the discrete emotional model, has mainly been used to explore basic emotions (i.e., sadness, happiness, fear, surprise, anger, and disgust; Baumgartner et al., 2006; Balconi and Mazza, 2009; Li and Lu, 2009). Attempts to differentiate tenderness from negative emotions through EEG features using machine learning (ML) algorithms have been made, as in the two studies conducted by Zhao et al. (2018a,b). In these studies, the authors found the frontal alpha asymmetry (FAA) and the midline theta power to be useful features to classify such emotional states.

Recently, a growing body of research investigated emotion-recognition with deep learning algorithms. Particularly convolutional neural networks showed promising results, outperforming traditional ML models in distinguishing emotional states (Aloysius and Geetha, 2017; Li et al., 2018; Moon et al., 2018; Yang et al., 2019; Zhang et al., 2020). However, these models are still in their infancy and have various limitations for EEG-based BCI classification due to the limited training data available (Lotte et al., 2018).

Here, we conducted a proof-of-concept study to establish a reliable method that can be later applied for EEG-based NFB on the self-regulation of complex emotional states. Therefore, we opted for a traditional ML algorithm, the linear Support Vector Machine (SVM), which is characterized by high generalization properties, low computational capacity, and sufficient validation in real-time application. We extracted both FAA and time-frequency, together with correlational features across the whole scalp. We tested the model's performance and generalization properties by applying a trial-specific cross-validation procedure. Furthermore, we applied a feature-ranking algorithm to reduce our dataset's dimensionality and to investigate which channels and frequency bands were the most discriminative between the two emotional states. We hypothesized that tenderness and anguish have different electrophysiological correlates, not reducible just to the FAA and that they can be distinguished using an ML approach. Finally, we calculated Spearman's correlation coefficient between subjective ratings and subject-specific classification accuracy to understand whether the SVM performance was associated with the self-evaluation of the emotional intensity, the mantras' utility, the fatigue, and concentration levels that subjects felt throughout the experimental blocks.

## 2. Materials and Methods

### 2.1. Participants

We recruited 14 healthy adults or young adults (7 males and 7 females) with no history of psychiatric disorder or neurological illness for this proof of concept experiment. Before analysis, we discarded EEG recordings from three participants (2 males and 1 female) due to the presence of excessive artifacts. The average age of the 11 remaining participants was 27 years (SD = 7), ranging from 19 to 46. All participants had normal or corrected-to-normal vision and had Brazilian nationality, except one German participant who fluently spoke Portuguese.

### 2.2. Emotion-Inducing Stimuli

**Figure 1:**
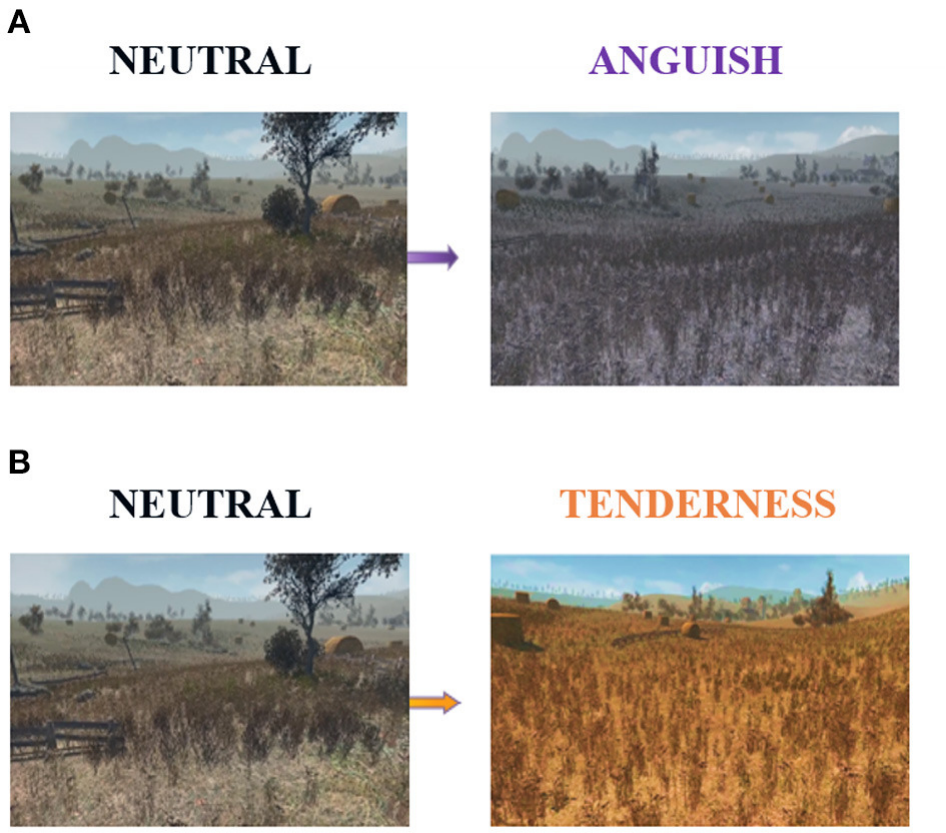
The virtual reality scenario, which we used to induce emotions during the experiment. Compared to the neutral-colored scene on the left, the color hue of the landscape turned purple **(A)** for anguish trials, while for all tenderness trials, it turned into a vivid orange **(B)**.

We implemented a multimodal stimulation with a virtual scenario, an edited version of the Unity 3D asset Autumnal Nature Pack, accompanied by emotion-specific musical excerpts. The virtual environment displayed the first-person view over a landscape of hills and cornfields, differently colored according to the emotion elicited: purple for anguish and orange for tenderness (Figure 1).

Together with these visual stimuli, participants listened to eight different instrumental musical excerpts of 46 s. Music excerpts were fixed for each trial type (i.e., 4 for anguish and 4 for tenderness) and normalized with the root mean square feature on the software Audacity (Audacity, http://www.audacityteam.org). For all tenderness trials, we played a piece of soft, harmonic, and gentle music. In contrast, we electronically manipulated the anguish trials; unpleasant stimuli from the originally pleasant tunes used for the tenderness condition. We used four new sound-files, in which the actual melodic excerpts were simultaneously played with two pitch-shifted versions of the same excerpts. The pitch-shifted versions were one tone above and a tritone below the original pitch (samples of the stimuli are provided at http://www.stefan-koelsch.de/Music_Emotion1), resulting in dissonant and distorted music. For the neutral condition, the landscape was typically colored, and no background music was presented.

### 2.3. Experimental Procedure

**Figure 2:**
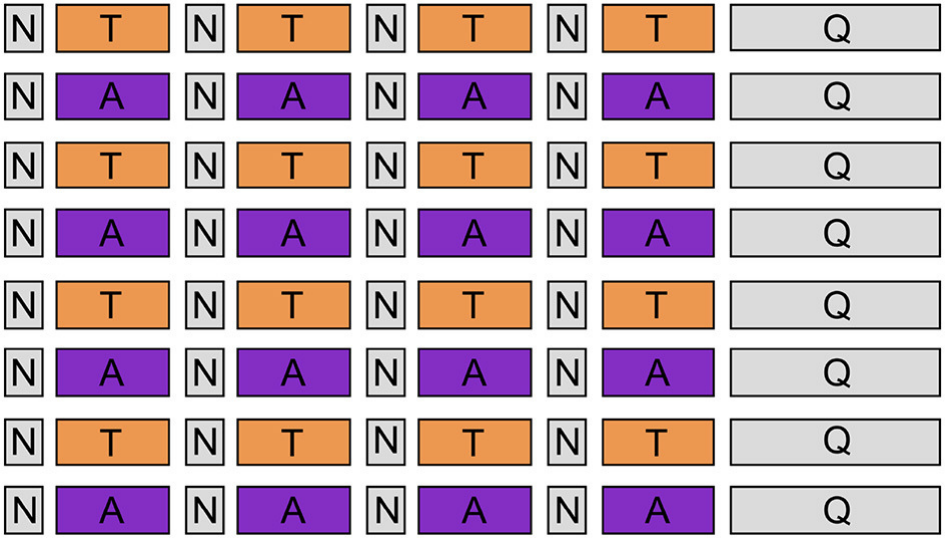
Experimental design. Eight emotion-alternating blocks (orange boxes refer to “tenderness” condition, and purple boxes to “anguish”), each consisting of 4 emotion-inducing trials of 46 s, interleaved by 4 short neutral trials of 12 s. At the end of each block, participants had to fill out a self-assessment questionnaire.

Upon arrival, all participants signed the informed consent and filled up the State-Trait Anxiety Inventory (STAI), the Beck's Depression Inventory (BDI), and The Positive and Negative Affect Schedule (PANAS). After the EEG cap setup, we instructed participants to use mantras and personally recalled memories to facilitate emotional self-induction during the experiment. As guidance, we provided a list of suggested mantras (Table 1), but they were free to choose mantras personally. Throughout the experiment, subjects were comfortably seated in an armed chair approximately 50 cm away from the screen, wearing padded headphones. The experimental procedure consisted of 8 emotion-alternating blocks (4 for anguish trials and 4 for tenderness), as represented in Figure 2. Each block included four emotion-eliciting trials (46 s) interleaved by four short neutral trials (12 s), which allowed for a brief rest between each emotional induction. The auditory and visual stimuli used for each trial are the ones described in the previous section. We recorded EEG signals throughout the whole experiment, and we gave no break between the blocks.

**Table 1:** Samples of mantras used according to the experimental conditions.

**Tenderness**	**Anguish**	**Neutral**
Compaixão protege tudo	Não vou conseguir nada	A Terra é redonda
Compassion protects everything	I will not achieve anything	The earth is round
Sentir o cheiro de um bebê	Doenças afligem tudo	A grama está crescendo
The smell of a baby	Diseases afflict everything	The grass is growing
Harmonia está em toda parte	Violência está em toda parte	A mesa é marrom
Harmony is everywhere	Violence is everywhere	The table is brown
Amor está em toda parte	O mundo está cheio de ódio	A luz está acesa
Love is everywhere	The world is full of hate	The light is on

*Free translation from Portuguese to English is provided below each sentence*.

#### 2.3.1. Subjective Ratings

At the end of each emotional block, participants had to fill a short self-assessment questionnaire in which they had to rate on a 5-point Likert scale: (i) the emotional intensity (from 1 = very mild to 5 = highly intense), (ii) the utility of mantras (from 1 = not useful to 5 = very useful), (iii) the concentration and (iv) the fatigue levels (from 1 = very low to 5 = very high) they felt throughout the experimental block.

### 2.4. EEG Acquisition

EEG data were recorded using the Brain Vision Recorder software. The signal was acquired from the BrainAmp amplifier and the standard 64 channels EEG-MR Plus cap (Brainproducts, Germany) at a sampling rate of 1,000 Hz and a bandwidth of 0.01–250 Hz. All the electrodes had sintered Ag/AgCl sensors placed according to the standardized international 10/20 EEG placement system, and their impedance was kept under seven kΩ. We used one of the 64 channels to record the ECG signal, and we set channel FCz as the reference electrode and channel AFz as the ground. We programmed the entire processing pipeline on Matlab (The Mathworks, Inc.).

### 2.5. EEG Preprocessing

We performed the offline analysis of EEG data using the EEGLAB toolbox (Delorme and Makeig, 2004) on Matlab R2019b (The Mathworks, Inc.). First, the preprocessing pipeline included downsampling the signal to 250 Hz and applying a bandpass Butterworth filter of 0.01–45 Hz. The Independent Component Analysis (ICA) algorithm was used to correct for eyeblinks and muscular artifacts. Components that captured artifacts were manually pruned independently for each subject. After artifact removal, the EEG dataset was epoched and cut into three distinct datasets for each subject according to the experimental condition. For each participant, we created one dataset for the 16 tenderness trials of 46 s length each, one for all the 16 anguish trials of 46 s length each, and one containing all 32 neutral trials of 12 s length. Data were then visually inspected, but we did not apply any further artifact or epoch rejection method. This choice was based on our future goal of using a similar setup for real-time neurofeedback studies, which will preclude the possibility to inspect and reject bad epochs visually. Therefore, we pursued a method allowing for effective classification despite the presence of some residual artifacts. Finally, we applied the surface Laplacian transform by implementing algorithms (Cohen, 2014) inspired by the spherical spline method described by Perrin et al. (1987a,b, 1989). This spatial filter allows reducing volume conduction effects for connectivity analysis purposes.

### 2.6. Feature Extraction

#### 2.6.1. Time-Frequency Analysis

The EEG data were transformed into the time-frequency domain using Complex Morlet Wavelet convolution to preserve information about the temporal dynamics. The Complex Morlet wavelet (CMW) is a complex-valued sine wave tapered by a Gaussian window described by the following equation:

(1) $$CMW=e^{-t^{2}/2s^{2}}e^{i2\pi ft}$$

Where *e*^−*t*^^2^/2*s*^2^ is the real-valued Gaussian and *e*^*i*2π*ft*^ is the result of the Euler's formula combined with a sine wave (Cohen, 2019). The time *t* is centered with regard to the wavelet by taking −2: sampling rate:+2 to avoid phase shifts.

One of the benefits of the CMW convolution over other methods such as Short-time Fourier Transform or the Hilbert Transform is the Gaussian-shaped wavelet in the frequency-domain. However, the caveat when doing convolution with a Complex Morlet Wavelet is to accurately define the width of the Gaussian, here defined as *s*, a key parameter for determining the trade-off between temporal and spatial resolution of the time-frequency analysis (see Cohen, 2019).

The parameter *s* is expressed as *s* = *c* /2π*f*, where *c* denotes the number of cycles of the wavelet, which is dependent on the frequency *f* of the same. A narrower Gaussian with fewer cycles in the time domain leads to a high temporal resolution but reduced spectral precision, and vice-versa with a wider Gaussian. Therefore, we applied a variable number of cycles ranging from 3 up to 10, increasing as a function of frequency to have a balanced trade-off between temporal and spectral resolutions. Since we were interested in all frequency bands, we selected a range of frequencies going from 1 up to 45 Hz. After applying CMW convolution, we extracted the power from the coefficients, and we applied a decibel-baseline normalization. Considering that we were mainly interested in changes in spectral features from a neutral mental state to the two distinct emotional ones, we used all the neutral trials as a baseline. To increase the number of samples, we cut the time-frequency data of each trial with a sliding-windows approach. Each window was 1 second long with a half-second overlap, resulting in a total of 91 windows per trial.

Then, we calculated the average change in power compared to the neutral baseline for seven frequency bands (delta 1–4 Hz, theta 4–8 Hz, low alpha 8–10 Hz, high alpha 10–12 Hz, low beta 13–18 Hz, high beta 18–30 Hz, and gamma 31–45 Hz). Within each window and for all the 63 channels and the seven frequency bands, the features extracted were the mean power, the standard deviation of the mean, and the frontal alpha asymmetry (FAA). The FAA coefficients were calculated for the channel pairs Fp1-Fp2 and F3-F4 in both low-alpha (8–10 Hz) and high-alpha (10–12 Hz) bands. The resulting feature array consisted of 2,912 samples for both classes (1,456 for anguish and 1,456 for tenderness) with a total of 886 features.

#### 2.6.2. Amplitude Time-Series Correlation

After CMW, we extracted each channel's amplitude information for each frequency component in the range of 1-45 Hz. We then applied the sliding-window approach again. Within each of the 91 windows and for each of the seven frequency bands aforementioned, we calculated the Spearman correlation coefficient of the 63*63 channels matrix. This time-frequency correlational analysis allows characterizing both the time-varying and frequency-varying properties of non-stationary signals such as electrophysiological signals. To eliminate redundant information and reduce the feature array's size, we only selected all the coefficients in the upper triangle of the correlational matrices. The resulting high-dimensional feature array consisted of 13,671 features (pairwise channel correlation coefficients for each frequency band) and 2,912 windows as samples for both emotional classes.

### 2.7. Classification and Feature Selection

We used the MATLAB R2019b Statistics and Machine Learning Toolbox for classification and visualization of the data. We opted for a linear Support Vector Machine (SVM) algorithm for high-dimensional data for binary classification of the feature arrays. SVMs are supervised learning algorithms that define a hyperplane as a decision boundary, such that the margin of separation between two classes is maximized. Herewith, SVMs provide a measure that allows scaling the certainty to which a window sample is assigned to one of the two classes: the sample's distance from the separating hyperplane. Regarding future applications in NFB, SVM allows for scaling the feedback (e.g., gradually changing color hue) and, thus, more precise response to emotional intensity changes.

We applied 8-fold cross-validation to train and validate the classifier. Therefore, we assigned all windows of one trial to the same fold. Thinking about possible future applications in NFB studies, we chose this method to estimate event-specific markers' impact on classification accuracy. As the model will be trained before the NFB session, we aimed at testing its performance on an entirely unknown set of trials. The four trials' windows (2 tenderness, 2 anguish) were thus kept as test sets, while the classifier was trained with the remaining 28 trials. We iterated the 8-fold cross-validation ten times and averaged across classification runs.

To visualize the datasets and understand the variability in performance across participants, we used t-Distributed Statistic Neighbour Embedding (t-SNE) (Van der Maaten and Hinton, 2008). T-SNE aims to preserve the local and global data structure when plotting all samples in a two-dimensional plane. The high-dimensional data is converted into a set of pairwise similarities and embedded in two dimensions such that similar samples are grouped together (Van der Maaten and Hinton, 2008). We applied t-SNE to the feature sets (time-frequency/FAA and correlational features) of the participant with the best and the one with the worst performance and tested whether it could succeed in finding two global data clusters, separating the two emotions.

Due to our dataset's high dimensionality, we performed feature selection through a feature-ranking algorithm using the Bioinformatics Toolbox of MATLAB 2019b. We aimed to improve the classifier's learning performance and identify the most common discriminative features across participants. Feature extraction and selection methods, besides improving predictive power, also help lower computational complexity and build models with better generalization properties (Liu et al., 2005; Anthony and Ruther, 2007). Using the *t*-test as an independent criterion for binary classification, we ranked the features after their significance between the classes. MATLAB's built-in function determines the absolute value of the two-sample *t*-test with pooled variance estimate for each feature. Essentially, the algorithm estimates how unlikely it is for a given feature that the difference in both emotional states' mean and variance occurred by chance. We set up two new feature arrays for each participant, using only the first 100 features selected by the feature-ranking algorithm. We classified the emotional states with the reduced sets of features using the abovementioned 8-fold validation method to test performance improvements. Furthermore, we extracted the 20 highest-ranked features for each subject to evaluate features' re-occurrence across participants.

#### 2.7.1. Statistical Comparisons

We performed statistical analysis of accuracy distributions using the Wilcoxon rank-sum method in order to understand which type of features performed better. We also wanted to investigate whether the SVM performances were significantly above chance, thus we statistically compared accuracy distributions of real-labeled data with surrogate data (i.e., randomly shuffled labels). Finally, we computed Spearman's correlation coefficients between participants' self-assessment questionnaire and SVM accuracy to assess whether there was a relationship between subjective ratings and differences in performance.

## 3. Results

First, we investigated through ML whether the two complex emotional states could be accurately differentiated in a subject-wise manner using time-frequency/FAA and correlational features extracted within 1 s windows. To ensure that classification performance did not depend on the high feature-to-sample ratio, we performed a feature-ranking procedure and we repeated classification with a drastically reduced number of features. Furthermore, we aimed at comprehending the inter-subject variability by visually inspecting the data distribution of individual subjects by using the t-SNE method. Then, we investigated the common highest-ranked features across subjects to shed light on the spatial and spectral properties that allowed discriminating between tenderness and anguish. Finally, we explored whether subjective emotional experience (i.e., self-ratings) showed any association with subject-dependent classification performance.

### 3.1. Classification Results Using SVM

**Figure 3:**
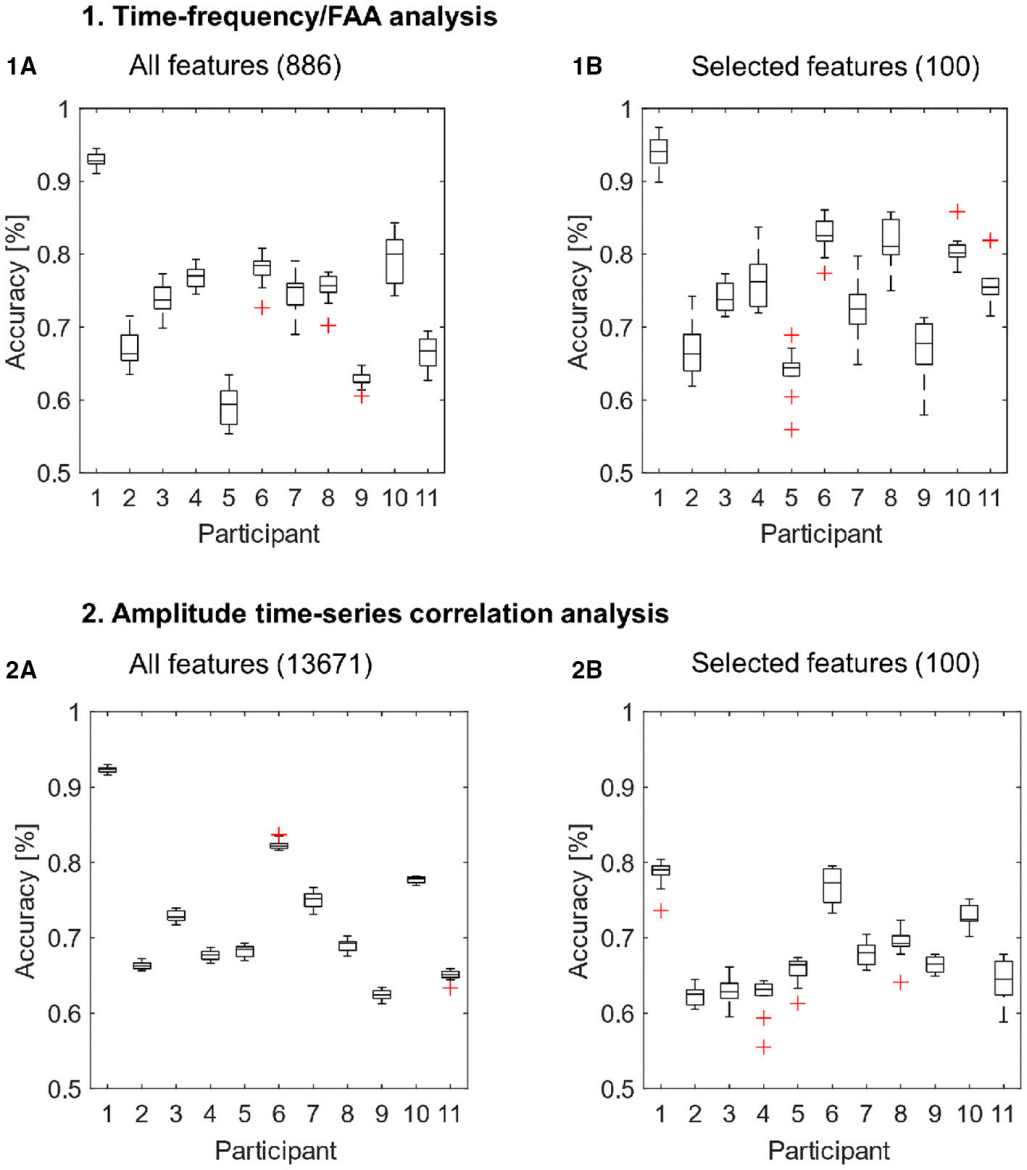
Classification results for the features extracted with the time-frequency/FAA analysis **(1A,1B)** and amplitude time-series correlation analysis **(2A,2B)**. The classification was performed with all available features **(1A,2A)** and exclusively with the 100 selected features **(1B,2B)** for each feature extraction method. We performed eight-fold cross-validation ten times, such that the boxplots depict the results of 10 classification runs for each participant.

The results of all cross-validation runs for each participant are presented in Figure 3. Corresponding average accuracies for each subject are summarized in Table 2. Furthermore, we compared the accuracy distributions for both types of features or feature arrays (i.e., whole features sets and subsets of selected features) using the Wilcoxon rank-sum test, reporting corresponding *p*-values.

**Table 2:** Classification accuracy in percent for each participant, feature extraction method, and the number of features.

**Participant**	**Time-frequency analysis and FAA**		**Amplitude time-series correlation analysis**	
	All features (886)	Selected features (100)	All features (13,671)	Selected features (100)
1	92.9	94.1	92.4	78.4
2	66.9	66.7	66.3	62.3
3	73.9	74.2	72.9	63.0
4	76.9	76.8	67.7	62.2
5	59.2	63.8	68.3	65.7
6	77.9	82.5	82.4	76.9
7	74.4	72.5	75.1	68.0
8	75.4	81.5	69	69.3
9	62.7	66.6	62.4	66.4
10	79.5	80.6	77.7	73.0
11	66.4	76.3	65.0	64.4
Average accuracy	73.28 ± 9.27	75.97 ± 8.72	72.65 ± 8.81	68.15 ± 5.69

*As we performed eight-fold cross-validation ten times, each result in the table represents a mean of the cross-validation runs. All accuracy distributions were significantly above chance level (p < 0.0001)*.

For the whole feature array extracted with the time-frequency/FAA analysis, we reported accuracies ranging from 59.2% up to 92.9% (mean = 73.28, SD = 9.27) when applying cross-validation (Figure 3, Subplot 1A). We report a range of accuracies from 62.4 to 92.4% (mean = 72.65, SD = 8.81) for the entire feature set extracted with amplitude time-series correlation analysis (Figure 3, Subplot 2A). The SVM performance was highly above chance-level (i.e., compared to surrogate data) for all participants using both time-frequency/FAA and amplitude correlation features (*p* < 0.0001). These results show that it is possible to discriminate between anguish and tenderness within-participants, despite the presence of a high inter-subject variability across both feature sets.

Then, due to the high-dimensionality of both feature sets, we performed a feature-ranking procedure and we repeated the 8-fold cross-validation with the selected subsets of 100 features. The subset of selected features significantly improved the model's predictive power for time-frequency/FAA features (*p* = 0.021; Figure 3, Subplot 1B). The results of 8-fold cross-validation using the 100 selected features through the feature-ranking algorithm yielded accuracies ranging from 63.8% up to 94.1% for the time-frequency/FAA feature set (mean = 75.97, SD = 8.72). In contrast, the performance significantly worsened (*p* = < 0.0001, Figure 3, Subplot 2B) for the selected 100 amplitude correlation features, with accuracies ranging from 62.2 to 78.4% (mean = 68.15, SD = 5.69). Nevertheless, the model's performance was highly above chance for both subsets of selected features (*p* = < 0.0001).

**Figure 4:**
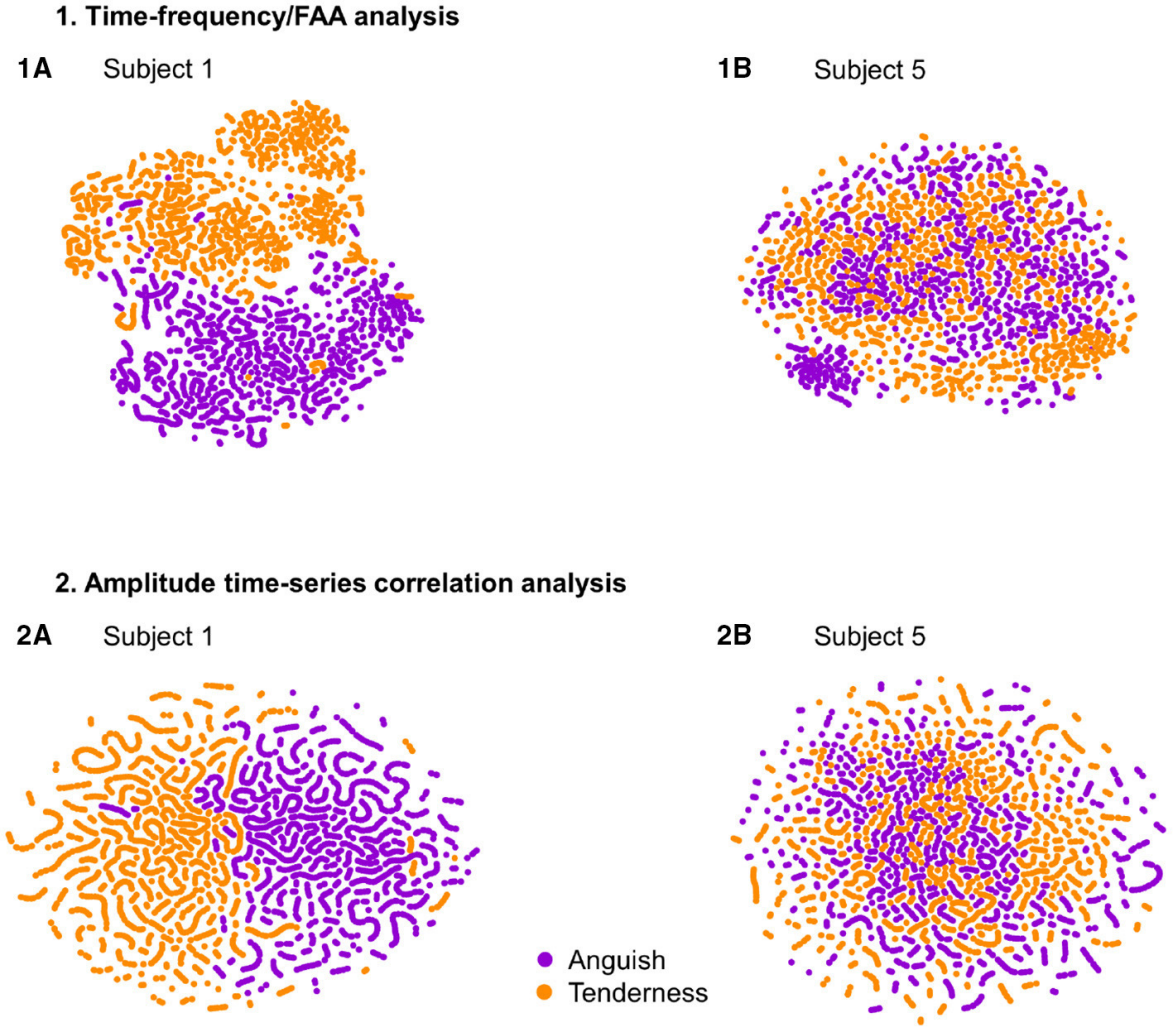
T-SNE visualization for the feature sets of subjects 1 and 5, with the best performance **(1A,2A)** and one of the worst classification performances **(1B,2B)**, respectively. Samples of the classes “Anguish” and “Tenderness” are plotted in violet and orange, respectively. The upper two plots **(1A,1B)** depict the time-frequency analysis datasets. The two plots at the bottom **(2A,2B)** display the two participants' amplitude time-series correlation features.

To understand the variability of performance across subjects, we plotted with t-SNE the datasets of participant 1 and participant 5, who had the best and one of the worst classification accuracies, respectively. The datasets' global geometry of subject 1 (Figure 4, Subplot 1A and 2A) can be separated into two clusters corresponding to the emotional states. In contrast, we could not find the same global structure for the datasets of participant 5 (Figure 4, Subplot 1B and 2B). Notably, the datasets resulting from the amplitude time-series correlation analysis (Figure 4, Subplot 2A and 2B) displayed a more defined regional pattern: samples clustering into lines. These clusters consisted of initially temporally adjacent samples. For the participant with the worst classification accuracy, participant 5, this regional pattern dominated the global data structure (Figure 4, Subplot 2B), suggesting that this phenomenon depends on temporal properties of connectivity rather than changes in neural correlation directly related to the emotional states.

### 3.2. Highest-Ranked Features Across Participants

**Figure 5:**
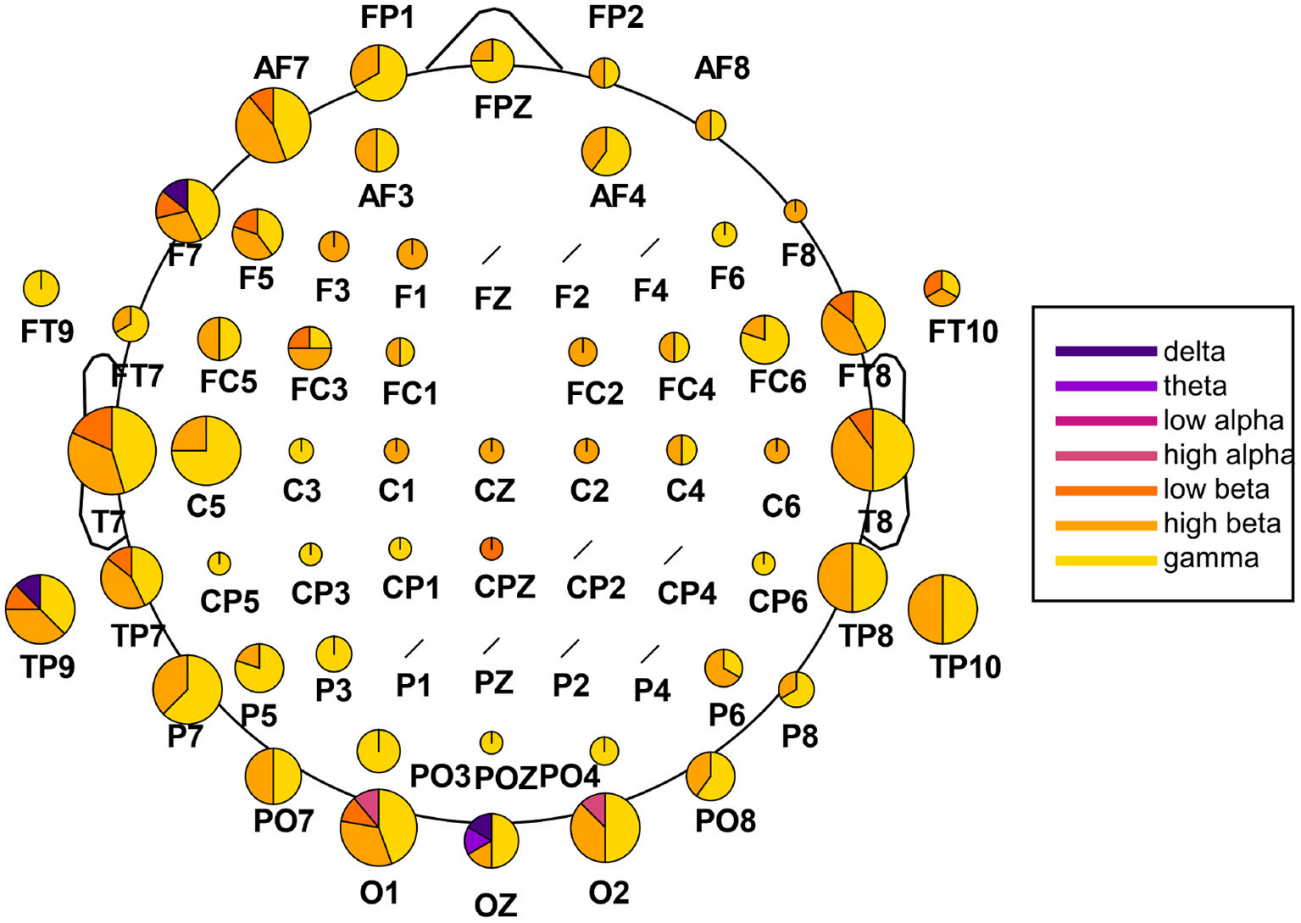
Extraction of the highest-ranked features for classification from the time-frequency analysis. The analysis was carried out across participants, with 220 features in total (20 per participant). The size of the channel-specific pie plot reflects how often the respective channel occurred in the main features. The pie plots' distribution indicates the relative amount of features from each frequency band,ranging from delta (dark violet) to gamma (yellow). Channels marked with a black slash did not occur in the main features.

We extracted the twenty highest-ranked features for each subject and each feature array in order to highlight which channels and frequency bands were the most discriminative between the two emotions. These main 220 features of the time-frequency/FAA analysis are presented in Figure 5. Notably, the FAA coefficients did not occur in this selection. Additionally, we summarized the features separated after brain region and hemisphere, irrespective of the frequency band, in Table 3. In the frontal site, we noticed a strong prominence of features in the left hemisphere compared to the corresponding right with a ratio of approximately 3:1 (35 to 11 features), when evaluating channels pairs Fp1/Fp2, AF3/AF4, AF7/AF8, F1/F2, F3/F4, F5/F6, and F7/F8. We showed the particular importance of channel AF7 compared to the complementary right-hemispheric channel AF8 and the strong dominance of gamma and high-beta bands in the highest-ranked features. Moreover, the extracted features showed the relevance of the occipital channels O1, O2, and Oz and both hemispheres' temporal sites.

**Table 3:** Extraction of the highest-ranked features for classification from the time-frequency analysis across participants separated after brain region and left/right hemisphere.

**Brain region**	**Left hemisphere**	**Total number of features**	**Right hemisphere**	**Total number of features**
	**Channel labels**		**Channel labels**	
Frontal	Fp1, AF3, AF7, F1, F3, F5, F7	35	Fp2, AF4, AF8, F2, F4, F6, F8	11
Temporal	FT7, FT9, T7, TP7, TP9	32	FT8, FT10, T8, TP8, TP10	36
Central - Parietal	FC1, FC3, FC5, C1, C3, C5,	39	FC2, FC4, FC6, C2, C4, C6,	20
	CP1, CP3, CP5, P1, P3, P5, P7		CP2, CP4, CP6, P2, P4, P6, P8	
Occipital	PO3, PO7, O1	19	PO4, PO8, O2	15
Midline	(FPz, Fz, Cz, CPz, POz, Oz)			13

**Figure 6:**
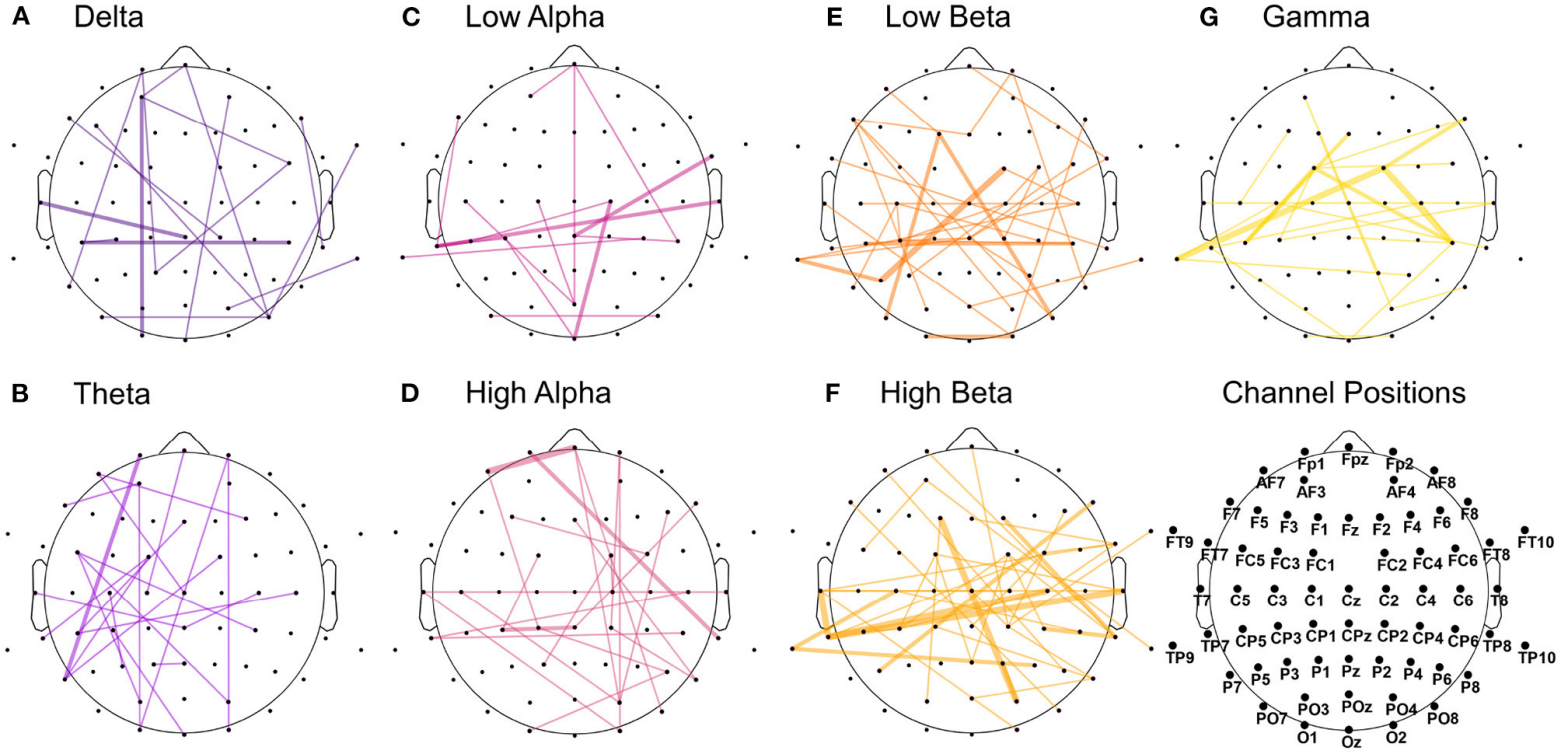
Features with the highest significance in distinguishing the emotional states from the amplitude time-series correlation analysis. The analysis was carried out across participants, with 220 features in total (20 per participant). The graph plots are separated into the seven extracted frequency bands, from delta (dark violet) to gamma (yellow). The connection's width reflects how often the correlation occurred in the main features across participants, with the thicker line representing higher occurrence across subjects. **(A)** Delta. **(B)** Theta. **(C)** Low alpha. **(D)** High alpha. **(E)** Low beta. **(F)** High beta. **(G)** Gamma.

On the other side, the highest-ranked features extracted from the amplitude time-series correlation analysis appeared to be more equally distributed across the seven analyzed frequency bands (Figure 6). In particular, theta and high-alpha ranges showed asymmetrical distribution in correlation, with a prominence in the left hemisphere for the former and in the right one for the latter. Significant correlations in low-alpha and beta, especially high-beta, appeared to be more prominent in the posterior part than the frontal one. The results also highlighted the importance of temporoparietal and temporo-temporal correlations in the beta and gamma bands (Figures 6E–G). In contrast, correlations in lower frequencies appeared to be dominant in the frontal and occipital sites (Figures 6A–D).

**Figure 7:**
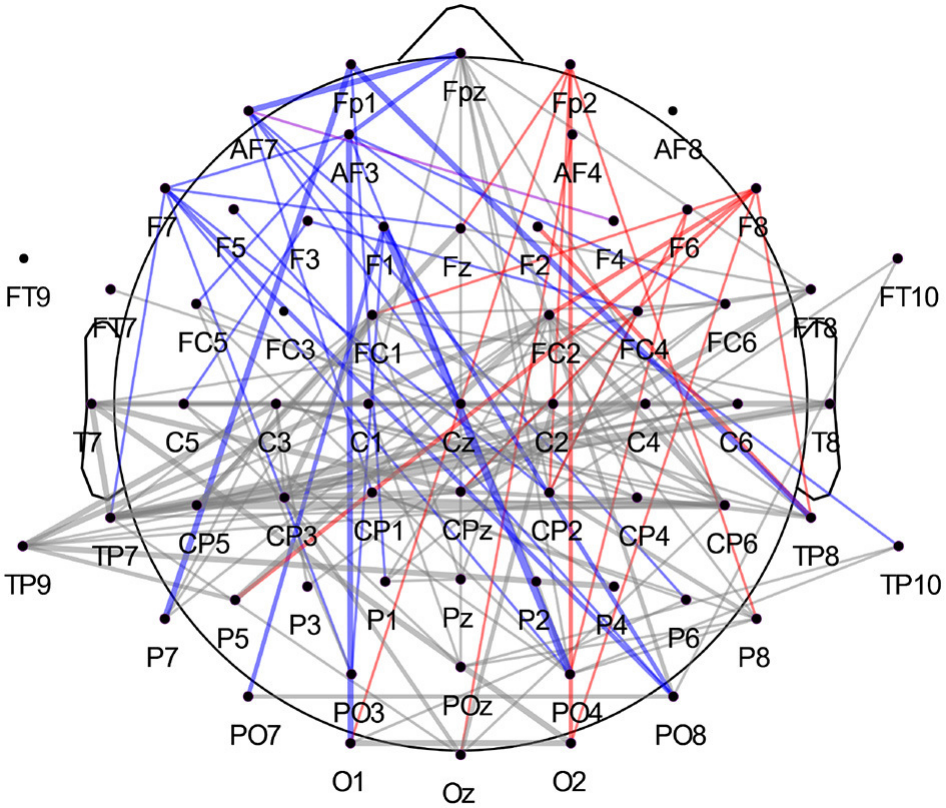
Highest-ranked amplitude time-series correlation features from all frequency bands. When separating correlations involving left frontal channels (in blue, Fp1, AF3, AF7, F1, F3, F5, F7) and right frontal channels (in red, Fp2, AF4, AF8, F2, F4, F6, F8), we observe a high prevalence of the left frontal cortex compared to the right. The analysis was carried out across participants, with 220 features in total (20 per participant). Again, the connection's width reflects how often the correlation occurred in the main features across participants, with the thicker line representing higher occurrence across subjects. The correlation between AF7 and F4 is plotted in violet and counted as both left- and right-hemispheric frontal correlation.

We observed that correlations involving the left frontal area occurred with more prevalence in the highest-ranked features across participants than correlations involving the right frontal hemisphere. The ratio was approximately 2:1 (44 to 20 features) when comparing channels Fp1/Fp2, AF3/AF4, AF7/AF8, F1/F2, F3/F4, F5/F6, and F7/F8. In Figure 7, we summarized all frequency bands and highlighted the correlations of these left and right frontal channels in blue and red, respectively. Once more, we report a high involvement of the left-hemispheric channel AF7 compared to the complementary right-hemispheric channel AF8, particularly in the high alpha band (Figures 6D, 7).

### 3.3. Subjective Ratings and Correlation With SVM Performance

**Figure 8:**
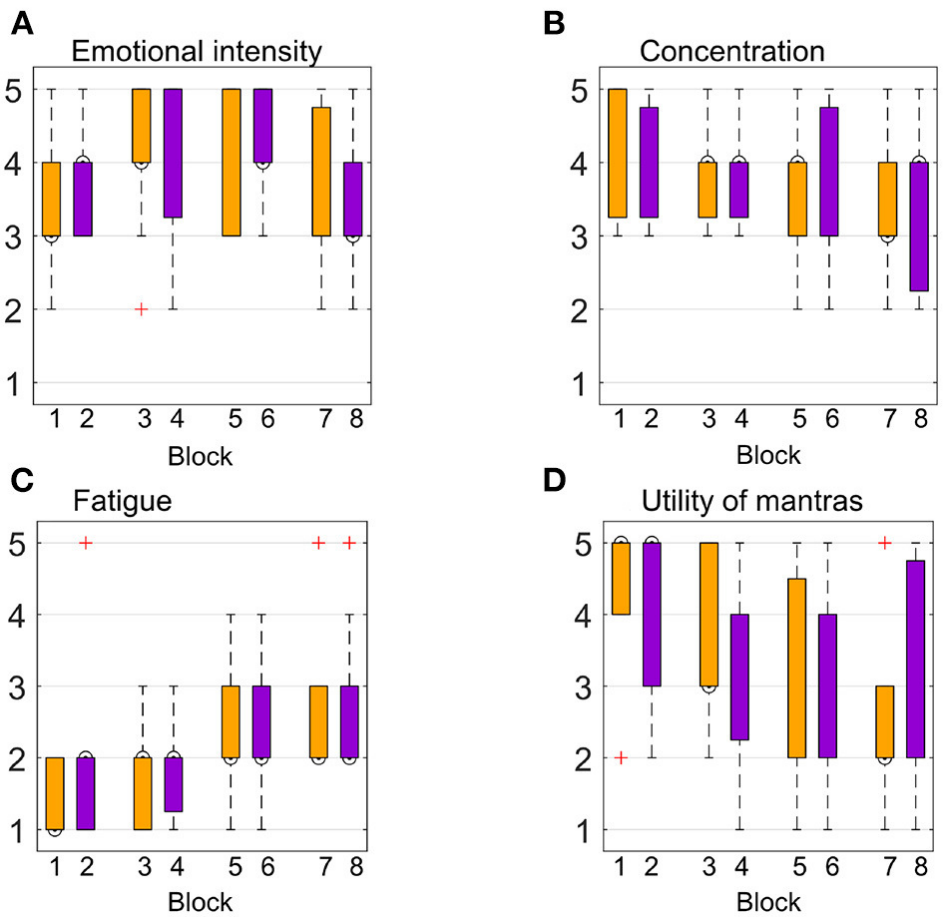
Participants' subjective ratings were assessed at the end of each of the eight blocks (four for each emotion). Orange refers to the “tenderness” condition, while purple refers to “anguish.” The y-axis represents the 5-point Likert scale, going from “very mild” to “very intense” for **(A)**, from “very low” up to “very high” for **(B,C)**, and from “not useful” to “very useful” for **(D)**.

We assessed emotional intensity, the usefulness of mantras, fatigue, and concentration levels at the end of each block. An overview of subjective ratings is presented in Figure 8. On average, the emotional intensity was rated “moderate” to “high” for the first and the last blocks, with a slight increase for the second and the fourth blocks. Figure 8A suggests that participants felt both tenderness and anguish with the same intensity. Concentration levels show minor differences across conditions and blocks, with the highest concentration level reported during the first block for both emotions. As expected, fatigue ratings showed a slightly increasing trend throughout the experiment, with a “very low” and “low” level for the first blocks and up to “moderate” levels for the last blocks. Average responses across participants with regards to mantras usefulness showed higher variance compared to the other self-rated measures across blocks and conditions, as shown in Figure 8D. Correlation analysis between subjective ratings and classifier performances using both feature sets was not significant for any of the four measures (*p* > 0.05).

## 4. Discussion

Our study demonstrated the feasibility of using EEG and ML tools to distinguish complex emotional states using a near-immersive emotion-elicitation paradigm. We selected stimuli, such as VR with music, that potentially serve as BCI for NFB experiments. We showed that, despite low spatial resolution and the inability to infer activity in subcortical structures, EEG is a suitable tool for classifying discrete emotions, such as tenderness and anguish. We performed cross-validation using SVM and we reached moderate to high accuracy using time-frequency/FAA and correlational features calculated in short time-windows. Furthermore, we were interested in identifying the most relevant features for classification that were shared among participants. To do so, we performed a feature-selection procedure and we repeated cross-validation using a subset of 100 selected features. We found that the most discriminative features belonged to channels of the frontal left hemisphere, of the temporal, and the occipital lobes. The feature-selection procedure also highlighted the importance of high-beta and gamma bands to distinguish between anguish and tenderness trials. Finally, we calculated the Pearson correlation coefficient between subjective ratings (i.e., the intensity of emotion, the utility of mantras, fatigue, and concentration levels) and classification performance across participants to investigate the relationship between the self-reported ratings and inter-subject variability in SVM performance. We reported no significant association between self-assessment measures and accuracy distributions.

One crucial challenge of neuroimaging studies on affective processes has usually concerned participants' difficulty in engaging and sustaining valid emotional states in an experimental setting, especially when trying to elicit complex emotions. Here, the use of a multimodal realistic virtual environment (VR) may have eased and encouraged the participants' involvement in the experiment by providing an engaging setup, as proven by previous studies (Cho et al., 2002; Lécuyer et al., 2008; Kim et al., 2014; Kovacevic et al., 2015). Moreover, musical excerpts' accompaniment may have facilitated emotion-elicitation, given that music is a powerful tool for inducing strong emotional experience (Brown et al., 2004; Trost et al., 2012; Koelsch, 2014). Lastly, the self-induction of emotional states through personalized mantras may have influenced the good outcome of discrete emotion classification, since we combined external elicitation with an internal locus of emotional induction. However, this may also have played a role in the substantial inter-subject variability in classification accuracy, given that self-reported ratings showed high variance in participants' responses to the mantras' usefulness. Nonetheless, it has to be noted that we did not find any significant correlation between any self-ratings measures and model's performance, suggesting that subjecting ratings were not associated with higher/lower performance.

Using time-frequency and FAA features, we reported accuracies ranging from 59.2% up to 92.9% when testing the model on unseen data. Similarly, features extracted from the amplitude time-series correlation analysis showed a comparable model performance when tested on unknown data, with the lowest accuracy being 62.4% and the highest 92.4%. The non-ergodicity in human subjects studies can explain the high interindividual variability we found in these results (Fisher et al., 2018). Several studies have observed individual differences in emotional processing (Canli, 2004; Aftanas et al., 2006; Kuppens et al., 2009; Molenaar and Campbell, 2009), stressing the importance of analyzing data at the individual level. Moreover, we assume that different amounts of noise in the datasets may have contributed to the inter-subject variability of classification results. As stated in the section 2, we decided not to apply any artifact removal or epoch rejection going beyond ICA to remove eyeblink and muscular artifacts. We wanted to test the classifier's performance as close as possible to the practical NFB scenario, in which epochs cannot be rejected during online analysis. We prioritized our model's utility in real-world application and aimed for a robust classification, despite the inclusion of noisy window-samples and a conservative validation with samples from unseen trials. Notwithstanding, reducing the datasets' dimensionality through a feature selection procedure led to lower performance variability across subjects, especially for correlational features.

Analysis of the highest-ranked features across participants did not show the importance of FAA features, as found in previous classification studies involving tenderness (Zhao et al., 2018b). However, as shown in Figure 5, the left frontal electrodes, especially channel AF7, appeared to be particularly discriminative for the two emotions. The relevance of the electrode AF7 is in line with previous studies using MUSE EEG headband comprising four channels (TP9, AF7, AF8, TP10) for emotion classification purposes, highlighting the importance of channel AF7 to accurately distinguish between mental states (Bird et al., 2019; Raheel et al., 2019). Interesting to notice is that channels from the right frontal hemisphere did not appear to be as discriminative as their left-sided counterparts, both for time-frequency and correlational features, as shown in Table 3 and Figure 7, respectively. The most common features across participants and both feature sets also revealed the relevance of temporal and occipital sites to discriminate between tenderness and anguish. We are not the first to report the importance of the temporal lobes for distinguishing between emotions. Zheng and colleagues conducted a study aimed at finding stable electrophysiological patterns across subjects and sessions of emotion recognition (Zheng et al., 2017). The authors found a stronger activation of lateral temporal areas for positive compared to negative emotions, both in the beta and gamma bands. Subject-independent features stemmed mostly from those brain areas and frequency bands (Zheng et al., 2017). Similarly, Jatupaiboon et al. (2013) found that the temporal lobe was particularly useful for classifying happy from unhappy emotions. Moreover, activation of sensory areas, such as temporal and occipital lobes, has been linked to emotional processing, regardless of the stimulus type. For example, in several neurological and psychiatric disorders, the temporal cortex's abnormal structure and function have been linked to emotion processing difficulties. Patients with temporal lobe epilepsy show deficits in emotion recognition, particularly fear and other negative emotions (Monti and Meletti, 2015). In schizophrenic patients, Goghari et al. (2011) found a correlation between reduced temporal lobe gray matter and poor facial emotion recognition (Goghari et al., 2011). Similarly, in frontotemporal dementia, a core problem is the impaired processing of emotional signals (Marshall et al., 2019). A different neuronal response has been observed in the occipital cortex in response to stimuli eliciting distinct emotions. Kragel et al. (2019) succeeded in predicting 11 specific emotion categories with convolutional neural networks, solely based on the occipital cortex's activity. Mattavelli et al. (2016) hypothesize that the frontal cortex exerts an early influence on the occipital cortex for discriminating emotions when a subject is presented with emotionally loaded visual stimuli. Regarding the contribution of the different frequency bands, high-beta and gamma bands had the highest impact on discrimination between emotional states across subjects. This result is also consistent with the literature providing evidence of these bands' importance for distinguishing different emotional states (Glauser and Scherer, 2008; Li and Lu, 2009; Daly et al., 2014; Li et al., 2019). However, it has to be noted that the feature-ranking algorithm does not allow us to infer which of the two classes showed increased or decreased spectral power or amplitude-related coherence patterns.

Although conducted in an offline manner, we would like to emphasize that the present study is a proof of concept addressing the crucial aspect of the classification of discrete complex emotions in short time-windows using EEG signals. Here we did not aim at maximizing classification accuracy at the expense of applicability in real-time. Deep learning methods such as CNNs are to date unsuitable for an individual-based real-time EEG experiment, where training data is limited and EEG feature sets are high-dimensional and sparse. As a rule of thumb, these networks are recommended to be given 10x the number of samples as compared to parameters in the network (Miotto et al., 2018). Moreover, parameter tuning for large networks trough trial and error is both time consuming and impractical, and online re-training of deep learning algorithms still remains under research (Aloysius and Geetha, 2017). Therefore, we chose the SVM as a ML algorithm with a low computational cost and online re-training ability, requiring both little training data and time when compared to the above-mentioned methods. Moreover, we provided appropriate validation techniques given the methods usually employed in an online setting.

Affiliative emotions, such as tenderness, have been proven to play a central role in a range of psychological and social processes. Mental training of such emotions has been increasingly applied in clinical settings. For example, there is evidence that Compassion-focused Therapy (CFT) is helpful for a broad range of mental health and neurological issues (Gilbert and Procter, 2006; Germer and Siegel, 2012). Loving-kindness meditation training has also shown positive effects on psychological well-being for both clinical and non-clinical populations (Lutz et al., 2008; Neff and Germer, 2013). With the EEG being the preferred tool in the clinical environment, the findings of this work are significant because they contribute to advances in the field of biofeedback and non-invasive neuroimaging approaches. Our proof-of-concept study promotes the development of personalized EEG-based NFB training on the regulation of such complex emotions, which may have therapeutic potential in restoring balanced neural activity for those suffering from emotional disturbances.

However, this study presents some pitfalls. Most importantly, the small sample size and the non-randomization of trials across participants, together with the presence of repetitive stimuli. The usage of the same stimulus presentation with no variety between trials (i.e., the naturalistic landscape) may have influenced sensory processing and classification results. At the same time, using identical musical tracks for all subjects may have influenced participants' emotional elicitation differently due to variances in individual taste in music. For future work on emotion elicitation, choosing personalized musical tracks could improve individuals' induction of emotional states. Another interesting future perspective would be to evaluate whether the combination of visual and auditory stimuli is more efficacious in inducing the desired emotions than either one sensory modality alone. Moreover, the dominance of a sensory domain at evoking emotions could be investigated by exposing participants to either only visual or auditory or mismatched stimuli. Another limitation of our study, as already stressed above, is the high inter-subject variability we found in the two affective states' classification. Although we assessed emotional intensity at the end of each block, we do not have any fine-grained assessment of the duration of the emotional experience throughout the experiment. Since emotions, both at experiential and neurophysiological levels, cannot be reduced to constant patterns across individuals (Barrett et al., 2007; Kuppens et al., 2009), future studies should involve a bigger sample size in order to shed light both on the individual differences and the common characteristics of emotional dynamics. Future directions would be to test this experimental protocol and SVM model on the training of such emotions through EEG-based NFB, which can represent another tool for exploring these affective states and contribute to the development of personalized interventions.

## 5. Conclusions

This proof of concept study showed that a multimodal near-immersive emotion-eliciting scenario is a feasible approach to evoke complex emotions, such as anguish and tenderness, in a laboratory setting. We proposed a pipeline that may be applicable for EEG-based BCI and NFB applications that require real-time data processing. Our findings indicate that the SVM classification of discrete emotions using EEG signals is achievable in a subject-wise manner, using short time-windows as in an online experimental setting. We identified features from the temporal, occipital, and left frontal sites as the most discriminative between the two emotions. Remarkably, we found a different involvement of left frontal and right frontal features, particularly in channel AF7 compared to channel AF8. The high-beta and gamma frequency bands appeared to have a substantial role in differentiating between anguish and tenderness. Together our results suggest that the EEG is a suitable tool for classifying discrete emotions, and that the proposed pipeline may be implemented in real-time to enable EEG-based NFB.

## Data Availability Statement

The dataset analyzed and the codes used in this study are available upon reasonable request from the corresponding author.

## Ethics Statement

The studies involving human participants were reviewed and approved by D'Or Institute Ethics and Scientific Committee. The patients/participants provided their written informed consent to participate in this study.

## Author Contributions

ED: methodology, formal analysis, data curation, writing—original, draft, review, and editing. MW: formal analysis, visualization, validation, writing—original, draft, review, and editing. BM: conceptualization, software, and investigation. CT-C: conceptualization, investigation, and supervision. TB: conceptualization, funding acquisition, and project administration. GD: methodology and supervision. JM: funding acquisition, resources, and supervision. All authors contributed to the article and approved the submitted version.

## Conflict of Interest

The authors declare that the research was conducted in the absence of any commercial or financial relationships that could be construed as a potential conflict of interest.

## Publisher's Note

All claims expressed in this article are solely those of the authors and do not necessarily represent those of their affiliated organizations, or those of the publisher, the editors and the reviewers. Any product that may be evaluated in this article, or claim that may be made by its manufacturer, is not guaranteed or endorsed by the publisher.
